# Effects of daily consumption of wild blueberry on cognition and urinary metabolites in school-aged children: a pilot study

**DOI:** 10.1007/s00394-021-02588-y

**Published:** 2021-05-23

**Authors:** Katie Louise Barfoot, Geoffrey Istas, Rodrigo Pedro Feliciano, Daniel Joseph Lamport, Patricia Riddell, Ana Rodriguez-Mateos, Claire Michelle Williams

**Affiliations:** 1 School of Psychology and Clinical Language Sciences, University of Reading, Earley Gate, Whiteknights Road, Reading, UK; 2 Department of Nutritional Sciences, School of Life Course Sciences, Kings College London, London, UK; 3 Division of Cardiology, Pulmonology, and Vascular Medicine, University Duesseldorf, Duesseldorf, Germany

**Keywords:** Flavonoids, Blueberries, Cognition, Executive function, Metabolite, Children

## Abstract

**Purpose:**

Acute intervention with wild blueberry (WBB) has previously revealed positive cognitive and mood effects in typically developing children; however, it is unclear whether effects persist after daily supplementation. In addition, no data have been published exploring the metabolite profiles of children following berry consumption, to our knowledge. A study of this kind could provide insight into a mechanism of action for the cognitive and mood improvements observed previously in children. The aim of this pilot study was to assess cognitive performance and urinary metabolite concentrations in healthy 7–10-year-old children across a 4 week daily WBB drink intervention.

**Methods:**

This pilot study examined the effects of daily WBB consumption for 4 weeks (766 mg total polyphenols; 253 mg anthocyanins; equivalent to 240 g fresh blueberries per day) on cognition and mood in 15 healthy 7–10-year-old children. Polyphenol metabolites were measured in 24 h urine before and after the 4 week intervention.

**Results:**

Chronic WBB-related benefits were seen on cognitively demanding trials on the modified attention network task, a task measuring executive functioning. Specifically, the WBB group maintained significantly higher accuracy on incongruent trials (96%; SE 0.03) compared with placebo participants (85%; SE 0.03; *p* = 0.038) after the 4 week intervention, suggesting WBB was of most benefit on the more difficult aspects of the task. No significant WBB-related effects were observed on the auditory verbal learning task or the child’s version of the positive and negative affect schedule. Urinary metabolite analyses indicated significant increases in different metabolites in WBB and placebo groups after 4 week consumption.

**Conclusion:**

The research demonstrates 24 h WBB bioavailability in a child cohort for the first time with increases in urinary hippuric acid excretion during 2 week daily WBB consumption. This study highlights the importance of conducting a larger study in children investigating the mechanism of action behind cognitive effects using bioavailability data.

**Supplementary Information:**

The online version contains supplementary material available at 10.1007/s00394-021-02588-y.

## Introduction

Evidence highlighting the benefits of polyphenols on human health has been expanding across the last decade [1–5]. Specifically, research into polyphenol-rich blueberries has revealed cognitive benefits in healthy adults [6–10] and children [11–15]. Positive effects on mood have also been observed across healthy adult [16], child [17] and adolescent [18] cohorts. Collectively, polyphenols have important implications for maintaining cognitive function and mental health across the lifespan.

The mechanisms of action (MOA) underlying such benefits are not currently confirmed, and research has started to investigate biological changes that occur across the period that health, cognition and mood effects persist. Identifying specific metabolites that circulate in the body after blueberries have been ingested can ascertain bioavailability [19–21]. This is important as it may reveal the metabolites that are circulating at the time of observed health or cognitive effects, highlighting a window, where polyphenols may be distributed to areas of the body, such as the brain, helping to determine a MOA. The specific mechanisms behind the dissemination of metabolites to these areas requires further exploration. At present, there is minimal literature on whether polyphenol metabolites are able to cross the blood–brain barrier (BBB) in humans, although research has suggested polyphenols can enter the brain endothelium [22] and permeate the BBB [23, 24] in animal models and in *in-vitro* models [25, 26].

Anthocyanins constitute a large proportion of the flavonoids found in blueberries; however, their bioavailability as intact compounds is known to be very low [27]. Recent investigations have focused on the detection, distribution and quantification of a wide number of anthocyanin-derived phenolic metabolites coming from the degradation, chemical breakdown and gut microbial metabolism of anthocyanins in the body. These low molecular weight phenolic compounds circulate at much larger concentrations than the parent anthocyanins, and have been postulated to be the major compounds circulating in plasma up to 1 h post-berry consumption [21, 27–31].

Exploration of the acute vascular benefits of blueberry consumption indicates that certain metabolites (particularly hippuric, vanillic, benzoic and ferulic acids) are present in urine and plasma after blueberry consumption and may be responsible for the acute endothelium-related vascular improvements observed in healthy volunteers [19, 32, 33]. It is unknown whether this MOA may also contribute to previously observed acute cognitive and mood effects. In Rodriguez-Mateos et al.’s [33] human study, metabolomic analysis was carried out on 63 phenolic anthocyanin derivatives in plasma following acute (2 h; 11 g; containing 150 mg anthocyanins) and chronic (28 days; 22 g/day; containing 300 mg/day anthocyanins) WBB consumption. Measures of vascular endothelium function were also taken using flow-mediated dilation (FMD) techniques. Fourteen metabolites were significantly associated with acute FMD improvements, 21 metabolites with chronic improvements and 9 metabolites correlated with acute and chronic FMD effects. This suggests that there may be some similar metabolite excretions following acute and chronic WBB and indicates that acute and chronic effects may be mediated by the same vascular MOA.

To date, no data have been published investigating the plasma or urinary metabolite profile following berry consumption in children. A study of this kind could provide insight into a MOA for the cognitive and mood improvements observed previously in 7–10 years [11–15, 17]. Better understanding of WBB-related changes in polyphenol excretion from this experiment may inspire future trials to investigate when metabolites may be in circulation and when they may have the potential to affect cognitive function alongside vascular measurement. Additionally, the longer term effects of blueberry polyphenols on cognition and mood in children has not been investigated. Thus, this study is the first, to our knowledge, to measure cognitive performance and metabolite bioavailability in children following a chronic WBB regimen.

The aim of the current pilot study was to measure cognitive performance and urinary metabolite concentrations in healthy 7–10-year-old children across a 4 week daily WBB drink intervention. It was hypothesised that executive function, memory and mood scores may improve in individuals assigned to the WBB treatment, based on previous research [11–15, 17]. It was also predicted that WBB metabolites may increase in a similar pattern to those identified in adults by Rodriguez-Mateos et al. [19, 32, 33] and Feliciano et al. [21]. As a pilot study, the intention was to recruit a small sample of participants to assess feasibility for future trials.

## Methods

The research was reviewed and given a favourable ethical opinion for conduct by the University of Reading Research Ethics Committee (UREC 15/10, UREC 15/58) and was conducted in accordance with the Declaration of Helsinki and Human Tissue Act 2004.

### Participants

Seventeen participants aged 7–10 years (*M* = 8.48, SD 0.96) were recruited from local primary schools in the Berkshire area, UK. Two participants withdrew from the study due to time commitment making the total recruited number 15 (8 female). On conducting a post-hoc power analysis based on MANT accuracy cognitive scores, the achieved sample size of 15 resulted in a low power of 0.4 [*F*(2,26) = 3.37]. This should be kept in mind when interpreting results. No participant had any food-related allergies or other health conditions, e.g., diabetes, obesity, blood pressure, thyroid, kidney and liver diseases, or psychological diagnoses e.g., ASD, ADHD, which would have excluded them from the study. No participants were defined as having attentional deficits (from parent reports or CPT scores). Demographic data are presented in Table 1.

**Table 1:** Demographic data for placebo and WBB participants

	Placebo (*n* = 7)			WBB (*n* = 8)			
	Mean	SD	Range	Mean	SD	Range	*p* values
Age (years)	8.46	0.98	7.02–9.07	8.30	0.88	7.00–9.08	0.73
CPT^a^							
Omissions (%)	9.88	4.34	4.58–17.08	4.64	2.87	0.42–8.33	0.02*
Commissions (%)	65.71	20.79	40–93.33	52.92	16.76	23.33–73.33	0.21
BAS 3^b^	52.14	3.13	50–59	52	7.46	40–63	0.96
F & V intake^c^	3.67	2.22	0.33–6	4.43	1.12	2.67–5.33	0.43
Taste ratings^d^	9.57	1.13	7–10	5.75	2.82	4–10	< 0.01**
Gender (M:F)	4:3	–	–	3:5	–	–	–

^a^Continuous Performance Task; Omissions measured as % incorrect out of 240; Commissions measured as % incorrect out of 30

^b^British Ability Scale 3; measured against norm data[39]

^c^Habitual fruit and vegetable intake; measured as portions per day

^d^Placebo *n* = 15; WBB *n* = 15; Scores from 1–10

* indicates *p* < 0.05; ** indicates *p* < 0.01

### Design

**Fig. 1:**
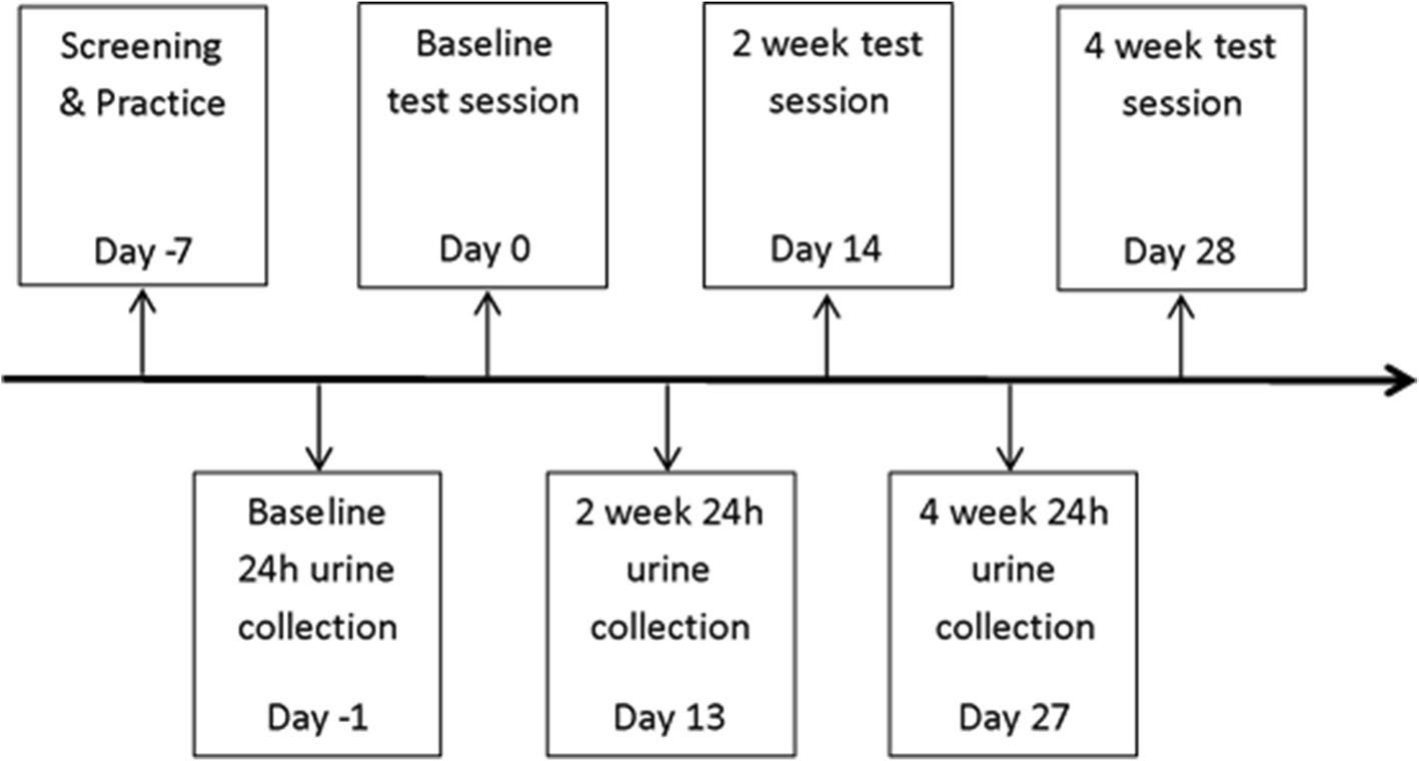
Between-groups study design comprising of mood, cognitive and urine data collection at baseline, 2 weeks and 4 weeks

In the current pilot study, children received a daily dose of either a 13.3 g WBB drink (766 mg total polyphenols; 253 mg anthocyanins; equivalent to 240 g fresh blueberries) or a placebo-matched control drink for 4 weeks using a between-groups, single-blind design. A single-blind design was utilised due to the researcher having to prepare and distribute the intervention and placebo sachets to participants. The researcher only saw drinks sachets upon distribution after the baseline test session and after the 2 week test session. Full blinding procedures were in place for analysis of the data. Measures of cognition (primary outcome) were recorded at baseline, 2 weeks and 4 weeks to ascertain the chronic effects of WBB on cognitive function and mood. Measures of polyphenol metabolite excretion (secondary outcome) were taken at baseline, 2 weeks and 4 weeks using 24 h urinary collections collected in the 24 h period prior to cognitive testing (see Fig. 1). Urinary excretions covering a 24 h period were used as they reflect the pool of circulating plasma metabolites in the body, from the small and large intestine. Using a 24 h pool of urine is more representative of chronic kinetics and better captures the half-life of metabolites in urine than a pool across a shorter timeframe [20, 34].

Testing took place after school (1530–1600), in keeping with previous child flavonoid research [15, 17] to minimise organisational demands and to maximise cognitive demand on participants.

### Treatments

Prior to screening, participants were randomly allocated to receive one of two treatment drinks; a wild blueberry drink or a matched placebo drink.

The WBB drink contained 253 mg anthocyanins and was prepared by mixing 170 ml water, 13.3 g of freeze-dried WBB and 30 ml of low-flavonoid Rocks Orange Squash. The placebo drink was matched to the WBB drink for sugars (4.79 g fructose, 4.52 g glucose), vitamin C (45 mg), Rocks Orange Squash (30 ml) and water (170 ml). Drinks were presented in an opaque cup and consumed through a black straw to maintain participant blinding procedures. Sufficient blinding was maintained when drinks were made at participant homes due to the experiment being advertised as a ‘fruit drink study’. This allowed both drink conditions to remain ambiguous, even when both drinks were tasted at screening. As a between-groups design was implemented, participants and their parents did not ever see both drinks contents.

Placebo and WBB interventions were weighed, packaged and stored at – 18 °C by a researcher in the University of Reading kitchen, and were distributed to participants as 2 × 14 daily sachets, alongside a 500 ml bottle of Rock’s Orange Squash, a 30 ml measurer, an opaque flask, straws and preparation instructions. Chronic procedures were in compliance with storage recommendations; all parents/guardians confirmed that the treatment sachets were retained in a freezer throughout the intervention period. All chronic interventions were prepared daily at the participants’ own homes by their parent(s)/guardian(s), and were consumed within 20 min of preparation. This was confirmed by drinks logs which stipulated the time the drinks were prepared and whether they were completely or partially consumed. In addition, there was no evidence that compliance was different between the groups. The time of daily consumption over the supplementation period was not stipulated for ease, due to the differing home schedules of children and their families.

### Test measures

#### Cognition and mood

All tasks used in the current study have been previously validated in our laboratory and have shown to be sensitive to nutritional interventions. Reys Auditory Verbal Learning Task (AVLT) assesses short-term verbal memory through word list learning, and requires participants to recall lists of 15 nouns after being presented audibly. Outcome measures for the AVLT include various sub-measures of verbal memory and interference, and were calculated according to Lezak [35]. In addition, implemented in the current study was a type of visual flanker task: the modified attention network task (MANT). The MANT measures executive function, attention and inhibition and produces outcome measures of accuracy (proportion of correct hits, 0–1) and reaction time (RT) for correct targets (RTs < 200 ms removed). Full details of both the AVLT and MANT can be seen in Barfoot et al. [15]. The Positive and Negative Affect Schedule for Children (PANAS-C) [36] was used as a measure of mood in the current study. This questionnaire asks participants to rate, on a scale of 1 (not at all) to 5 (very much), how much they feel each item (e.g., happy, miserable) in the current moment and is deemed suitable for children in the 7–10 age range. Higher scores indicated higher levels of positive or negative affect (for full details see [17]).

#### Procedure

Children were individually tested in a quiet cubicle space in the Nutrition and Cognition lab in the University of Reading Psychology Department on four separate visits—screening, baseline, 2 week and 4 week sessions.

#### Screening

All children took part in a screening session 1 week before the baseline test session. At screening, participants completed a computerised Continuous Performance Task (CPT) that was used to assess, but not diagnose, possible attentional deficits which may lead to exclusion from the study. Three sub-tests of The British Ability Scale 3 (BAS 3; matrices, pattern construction and verbal similarities) [37–39] were also administered to measure general cognitive ability. Seventeen participants were initially screened. Randomisation of intervention group membership was conducted using a random number generator prior to the screening session. Participant dropout occurred following screening (*n* = 1) and following baseline (*n* = 1); therefore, 15 participants completed the full study and had data sets analysed. All participants were of typical general cognitive ability for their age (Table 1). Participants did significantly differ between treatment groups on the CPT omissions subscale in that placebo participants made more errors of omission than WBB participants (Table 1); however, upon further exploration this did not appear to affect MANT or AVLT performance between groups at baseline.

A practice of the PANAS-C and cognitive task battery occurred at screening to ensure understanding of the tasks and to reduce practice effects [40]. A taste rating scale (10-point Likert scale ranging from ‘1—It was horrible’ to’10—I loved it’) was distributed to participants at the end of screening to assess their liking of the treatment drinks. Here, children were required to taste both treatment drinks in a randomised order. An exclusion criterion was applied, wherein any child who reported a rating of less than 4 for their assigned drink was excluded from the study. Four out of ten was chosen as a clear distinction between the children being indifferent to the drink (which a score of 5 out of 10 would represent) and actively disliking the drink. However, no child rated their drink less than 4 and so no child was excluded on this basis. Children were told that they would be randomly allocated to receive one of the two fruit drinks for the duration of the 4 week intervention. Urine collection and treatment procedures were explained to parents and children at screening.

Participants were required to follow a low-flavonoid diet for 48 h before each future test session. This consisted of participants avoiding high flavonoid foods, such as berry fruits, fruit juices, tea, coffee and cocoa. Low flavonoid suggestions were provided, such as potatoes, carrots, bananas and yoghurt. This 48 h period included the 24 h urine collections. Time of urinations and food consumption during 24 h urine collections were recorded by parents to check schedule adherence. Three day food diaries were used to record participants’ typical food consumption between baseline, 2 week and 4 week sessions (excluding low-flavonoid diet days). Food diaries were consistently completed on two weekdays and one weekend day, and these were used to assess habitual fruit and vegetable consumption (Table 1).

#### Urine collection procedure

Three urine containers (1 × 100 ml, 2 × 2L) were distributed to parents to collect their child’s fasted 0 h, 0–12 h and 12–24 h urine, 1 day prior to 0, 2 and 4 week test visits. Each 2L urine container contained 375 mg of L-ascorbic acid to preserve the urine across the collection period.

**Fig. 2:**
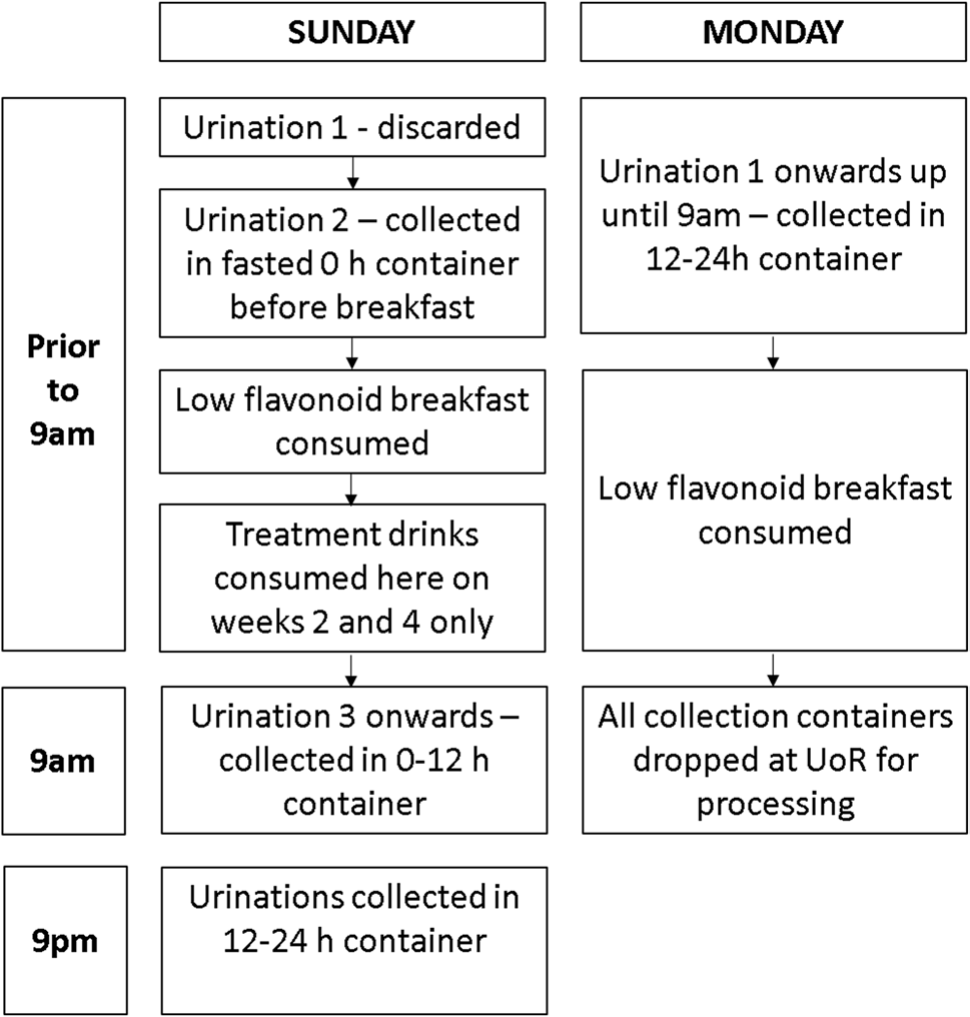
Urine collection day procedure for 0, 2 and 4 weeks into the intervention

Due to the level of continuous monitoring and support required of parents, urine collection days were encouraged to take place on a Sunday when both the child and parent were at home and ended Monday morning prior to school. Urine collection procedures across Sunday and Monday collection days can be seen in Fig. 2. The first urination of the day was excluded from urine collections due to the accumulation of compounds from the previous night. After the first urination of the day, participants were asked to use the fasted 0 h container to collect urine before consuming food. After this urination, participants were then permitted to start eating their (non-standardised) low-flavonoid diet for the day, and were instructed to use the 0–12 h container to collect each subsequent urination. The first urination in the 0–12 h container was permitted to be no later than 9am. Twelve hours after the initial 0–12 h urination, participants were asked to use the 12–24 h container to collect subsequent urinations. Parents completed the urine collection log detailing the time of the child’s first urination in each pot, and food consumed throughout the day, to ensure compliance to time and dietary requirements.

Participants were asked to store the urine containers in a cool bag with ice blocks (both provided) throughout the 24 h collection, and parents were encouraged to replenish with a new ice block every 3–4 h (excluding overnight). Following all collections, parents dropped their child’s samples at the University of Reading Psychology Department at 9am the following day, where they were processed and stored at –80◦C in the Food and Nutritional Sciences department.

During the second and third urine collections, participants consumed their assigned treatment drink immediately prior to commencement of the 0–12 h collection (no later than 9am). This was so comparisons could be made between the metabolites excreted across 0–24 h at 0 weeks (no treatment) and those excreted following WBB or placebo consumption at 2 and 4 weeks.

#### Urine processing procedure

Total volumes of baseline, 0–12 h and 12–24 h collection samples were recorded, and 100 ml of each respective container was retained for further processing after mixing. Samples were centrifuged and measured into 2 × 14 ml falcon tubes, where 1 × 14 ml falcon tube contained approximately 100ul formic acid (FA) to reach a pH of 2.4. One ml of urine from each falcon tube was then aliquoted into labelled Eppendorf tubes and stored at − 80 °C for future analysis.

#### Test day procedure

Baseline (week 0), 2 week and 4 week test sessions were conducted after school hours on a Monday, following urine collections on the Sunday. At the baseline session the completed 3 day food diary and urine collection log were retrieved from parents. Participants completed the PANAS-C and cognitive task battery before consuming either the placebo or WBB treatment drink. At the end of the baseline test session, the drinks-making procedure was explained to parents and children, and the necessary equipment was distributed. Neither parents nor children knew which treatment they were receiving. Parents continued supplementing their children at home with the same treatment that their child had received at the baseline test session over the next 2 weeks.

At the 2 week session, completed food diaries and urine collection logs were retrieved from parents. Children completed the PANAS-C and a different matched version of the cognitive task battery. Participants had not received a treatment drink on this day to avoid testing acute effects. Participants consumed their treatment drink at home after their test session and continued their treatment schedule for a further 2 weeks.

The same procedure ensued at the 4 week visit. At the end of this visit both the participant and their parent(s) were debriefed on the aims of the study and were reimbursed with £40 cash, a t-shirt and a cap for taking part.

#### Urinary analysis procedure

Urinary samples were analysed according to a validated method as described in Feliciano et al. [41]. The detection of urine polyphenol metabolites was performed on an Agilent 6550 iFunnel Accurate-Mass Quadrupole Time-of-Flight Mass Spectrometer (Q-TOF MS) through an electro-spray interface with Jet Stream technology after separation on a 1290 Infinity UHPLC system (Agilent, Waldbronn, Germany). Polyphenol concentrations were calculated using authentic standard curves. These curves were quantified within the Agilent software by calculating the mass of a metabolite compound and the time taken to elute within the gas chamber to produce a urinary concentration value. Recoveries were calculated by normalising urinary concentrations in terms of volume excreted during each collection timepoint. Twenty seven metabolites were quantified based on previous findings [20, 21].

### Statistical analysis

All data were analysed using SPSS (Version 22.0).

Fifteen participants (7 placebo, 8 WBB) were included in the analyses of demographic and mood variables. Baseline differences in age, attention (CPT; omissions, commissions), general ability (BAS 3) and taste (1–10) were examined using independent t tests with Drink (placebo and WBB) as the IV.

Mood (PANAS-C), cognitive (AVLT, MANT) and metabolite data were analysed by linear mixed models (LMMs) using an unstructured covariance matrix to model repeat measures. Separate Linear Mixed Models (LMMs) were performed for each dependent variable (DV) in the PANAS-C (PA, NA) and MANT (accuracy, reaction time). Separate LMMs were performed for each outcome measure of the AVLT, for each metabolite compound and for total polyphenols (sum of all compounds at each respective timepoint). Baseline performance was included in all LMMs as a fixed factor. For urinary metabolite analyses, within-group post-hoc comparisons were performed, following previously published methods [20, 21].

Drink (placebo, WBB), Time (2 weeks, 4 weeks) and Drink × Time were included as Fixed Factors in LMMs to compare the effects of treatment across the intervention period. For the MANT, Congruency (congruent, incongruent), Load (high load, medium load) and Target Time (120 ms, 500 ms) were also included as Fixed Factors in the model to detect changes in relation to cognitive load as in Whyte et al. [12, 13] and Barfoot et al. [15]. These variables were also contained within interactions with Drink and Time to assess cognitive load differences between treatment groups across the intervention. Participant was included as a random factor to accommodate the dependency of individual data. Fourteen participants (7 placebo, 7 WBB) were included in the analyses of MANT variables. This was due to corruption of a single participant’s MANT data file at the 2 week timepoint, rendering raw data void for this participant.

All post-hoc pairwise comparisons were corrected for type 1 errors using Bonferroni adjustment within each LMM.

## Results

### Cognitive results

#### Demographic data

Demographic data are presented in Table 1. There were no significant differences at baseline between treatment groups for age, inhibition (CPT commissions), general ability (BAS 3) or habitual fruit and vegetable consumption. There were significant differences at baseline between groups for sustained attention (CPT omissions), where placebo participants had a higher rate of failing to respond to non-target stimuli than WBB participants. There were also significant differences between groups for taste ratings, where drink ‘liking’ ratings were higher for placebo compared to WBB.

Raw data for cognitive and mood variables are presented in Table 2.

**Table 2:** Mean (SD) data for Placebo and WBB participants’ performance for mood and cognitive outcome variables at baseline, 2 weeks and 4 weeks

Outcome variables	Baseline			2 weeks			4 weeks		
	Placebo (*n* = 7)	WBB (*n* = 8)	*p*	Placebo (*n* = 7)	WBB (*n* = 8)	*p*	Placebo (*n* = 7)	WBB (*n* = 8)	*p*
Mood									
PA	51.86 (19.91)	54.63 (17.30)	0.78	49.29 (19.42)	52.63 (17.56)	0.73	46.57 (19.58)	49.38 (20.35)	0.79
NA	20 (12.83)	15.75 (0.89)	0.37	18.29 (6.21)	15.50 (0.53)	0.23	17.86 (7.56)	16.63 (3.29)	0.68
MANT accuracy (0–1):	(*n* = 7)	(*n* = 7)		(*n* = 7)	(*n* = 7)		(*n* = 7)	(*n* = 7)	
Overall	0.81 (0.11)	0.92 (0.04)	0.02	0.85 (0.17)	0.97 (0.02)	0.10	0.89 (0.12)	0.97 (0.01)	0.1
Congruent trials	0.85 (0.08)	0.96 (0.03)	< 0.01	0.89 (0.14)	0.98 (0.02)	0.13	0.91 (0.12)	0.98 (0.02)	0.19
Incongruent trials	0.77 (0.16)	0.88 (0.06)	0.1	0.82 (0.20)	0.96 (0.02)	0.08	0.86 (0.13)	0.96 (0.02)	0.06
High load trials	0.82 (0.12)	0.92 (0.05)	0.06	0.86 (0.15)	0.96 (0.03)	0.12	0.90 (0.10)	0.96 (0.02)	0.12
Medium load trials	0.80 (0.11)	0.93 (0.03)	0.01	0.85 (0.18)	0.98 (0.02)	0.09	0.87 (0.15)	0.98 (0.01)	0.09
Fast (120 ms) trials	0.79 (0.12)	0.90 (0.07)	0.07	0.83 (0.18)	0.96 (0.03)	0.09	0.86 (0.14)	0.97 (0.02)	0.06
Slow (500 ms) trials	0.82 (0.11)	0.95 (0.02)	0.01	0.87 (0.16)	0.97 (0.01)	0.12	0.91 (0.10)	0.97 (0.01)	0.18
RT (ms): overall	632.30 (104.28)	644.77 (93.81)	0.82	614.32 (95.05)	621.59 (80.53)	0.88	612.30 (83.86)	609.19 (66.09)	0.94
Congruent trials	612.64 (108.83)	596.45 (94.02)	0.77	598.17 (98.53)	603.92 (85.93)	0.91	600.57 (83.50)	589.15 (67.12)	0.78
Incongruent trials	651.95 (101.79)	693.09 (100.01)	0.46	630.46 (92.17)	639.25 (79.22)	0.85	624.02 (86.24)	629.22 (67.02)	0.9
High load trials	637.12 (106.45)	656.71 (92.14)	0.72	616.21 (97.28)	620.65 (79.08)	0.93	616.44 (91.83)	621.00 (68.37)	0.92
Medium load trials	627.48 (102.78)	632.84 (97.47)	0.92	612.43 (93.27)	622.53 (83.42)	0.83	608.16 (77.72)	597.38 (65.20)	0.78
Fast (120 ms) trials	601.88 (112.65)	647.93 (90.28)	0.42	617.59 (113.43)	623.69 (78.52)	0.91	597.39 (83.22)	598.59 (64.91)	0.98
Slow (500 ms) trials	662.71 (105.10)	641.62 (105.45)	0.71	611.04 (79.49)	619.49 (84.34)	0.85	627.21 (86.39)	619.79 (69.01)	0.86
AVLT	(*n* = 6)	(*n* = 8)		(*n* = 7)	(*n* = 8)		(*n* = 7)	(*n* = 8)	
Word span (A1)	3.50 (0.84)	3.63 (1.51)	0.86	4.14 (1.07)	4.00 (0.76)	0.77	4.00 (1.15)	4.13 (1.13)	0.84
				(*n* = 6)	(*n* = 8)		(*n* = 6)	(*n* = 8)	
Words learnt (A5–A1)	3.50 (1.87)	5.75 (3.24)	0.16	4.00 (1.67)	5.50 (2.33)	0.21	3.17 (3.66)	3.50 (2.20)	0.84
				(*n* = 7)	(*n* = 8)		(*n* = 7)	(*n* = 8)	
Final acquisition (A5)	7.00 (2.45)	9.38 (2.50)	0.1	7.57 (2.82)	9.50 (2.51)	0.18	6.71 (3.50)	7.63 (2.07)	0.54
Total acquisition (sum of list A: 1–5)	30.83 (9.85)	34.88 (6.56)	0.37	32.00 (9.00)	34.25 (7.96)	0.62	30.00 (9.88)	31.50 (6.65)	0.73
	(*n* = 7)	(*n* = 8)							
PI (A1–B1)	− 0.71 (1.25)	− 0.88 (2.17)	0.87	0.29 (1.38)	− 0.50 (1.20)	0.26	0.71 (0.76)	1.25 (2.12)	0.54
RI (A5–A6)	1.14 (1.95)	1.88 (2.47)	0.54	2.43 (3.26)	1.50 (1.20)	0.47	2.14 (2.61)	1.88 (2.53)	0.84
Total list A recall (sum list A: 1–7)	36.14 (18.89)	48.63 (8.53)	0.12	42.29 (12.84)	47.88 (11.73)	0.39	39.29 (14.28)	41.75 (9.79)	0.7
Total list recall (sum list A: 1–7 + B1)	39.86 (21.21)	53.13 (9.49)	0.13	46.14 (13.50)	52.38 (11.90)	0.36	42.57 (14.86)	44.63 (9.36)	0.75
Recognition	10.29 (2.36)	11.13 (1.89)	0.46	9.71 (2.50)	10.50 (2.07)	0.52	8.86 (2.79)	10.38 (1.69)	0.22
Long delay recall (A7)	4.86 (2.61)	6.25 (2.71)	0.33	5.14 (2.61)	5.63 (2.33)	0.71	4.71 (1.89)	4.50 (2.27)	0.85
Short delay recall (A6)	(*n* = 5)	(*n* = 8)		(*n* = 6)	(*n* = 8)		(*n* = 6)	(*n* = 8)	
	6.80 (2.17)	7.50 (1.31)	0.48	6.00 (1.26)	8.00 (2.73)	0.12	5.33 (2.73)	5.75 (2.76)	0.78

### MANT

Correlational analyses revealed that there was no trade-off between accuracy and reaction time (RT) scores at baseline (*r* = 0.26, *p* = 0.35).

#### Accuracy (proportion correct 0–1)

**Fig. 3:**
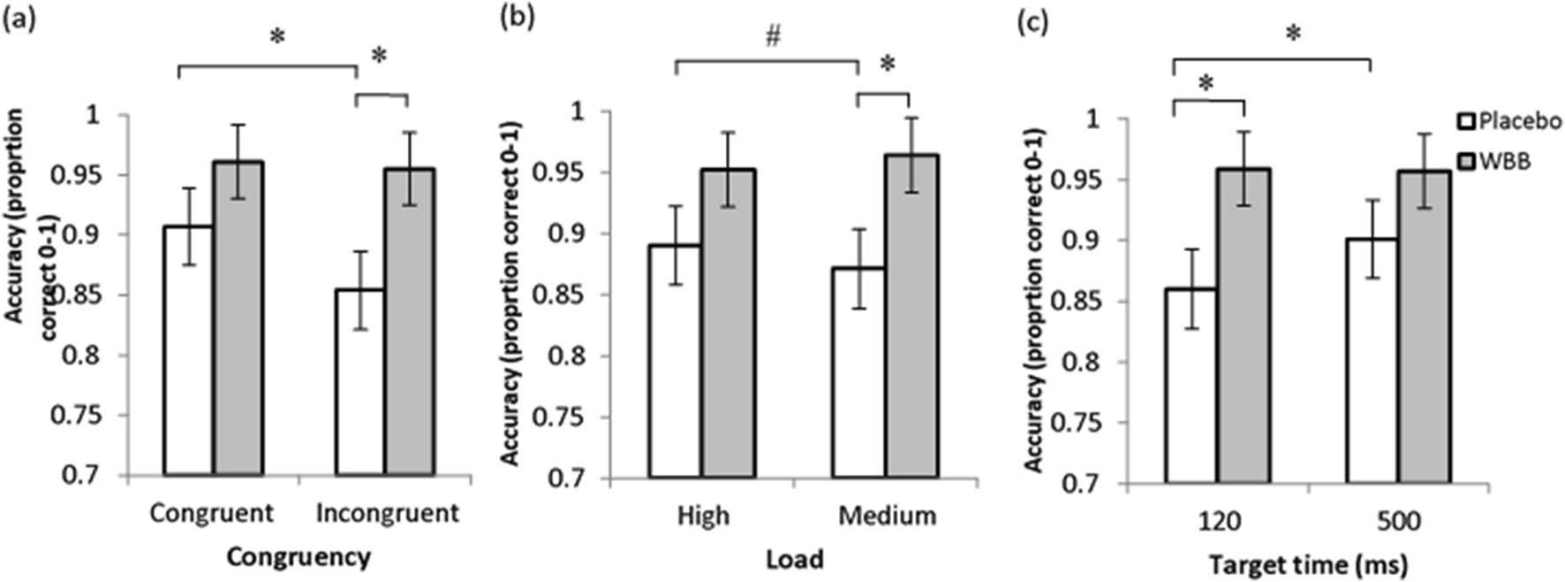
Mean (± SEM) MANT accuracy (0–1, proportion correct) for WBB and placebo participants on **a** congruency, **b** load and **c** target time variables; *Significance *p* < 0.05; # denotes a trend 0.05 > *p* < 0.1

Analyses of MANT accuracy scores showed three significant (*p* < 0.05) Drink interactions: (Drink × Congruency [*F*(1,104.15) = 10.74, *p* < 0.01], Drink × Load [*F*(1,104.38) = 4.84, *p* = 0.03], Drink × Target time [*F*(1,105.82) = 9.39, *p* < 0.01] as seen in Fig. 3.

Placebo participants performed at 91% accuracy on congruent trials which significantly fell to 85% on incongruent trials (*p* < 0.01). No such difference in performance was observed between congruent and incongruent trials for WBB participants, with participants performing at 96% accuracy on both incongruent and congruent trials. Indeed, WBB participants were found to be significantly more accurate on incongruent trials than placebo participants by 11% (*p* = 0.038; Fig. 3a.)

WBB-treated participants were also significantly more accurate than placebo on medium load trials (*p* = 0.05; Fig. 3b). A similar pattern was evident for high load trials, though this effect did not reach significance (*p* = 0.18).

As shown in Fig. 3c, significantly increased accuracy was also observed on 500 ms trials compared to 120 ms trials for placebo participants only (*p* < 0.01). The absence of between-trial differences in WBB participants (*p* = 0.87) suggests a maintenance of performance across different speeds of stimulus presentation within this group. WBB-treated participants showed 96% accuracy compared to 86% accuracy in placebo-treated participants on 120 ms trials (*p* = 0.04), highlighting the benefit of WBB on trials of faster presentation.

Regardless of treatment, accuracy was found to significantly increase at 4 weeks (*M* = 0.92, SE 0.02) compared to 2 weeks [*M* = 0.91, SE 0.02; *F*(1,119.71) = 5.34, *p* = 0.02], participants performed significantly more accurately on congruent trials (*M* = 0.93, SE 0.02) than incongruent trials [*M* = 0.91, SE 0.02; *F*(1,106.44) = 13.39, *p* < 0.01], and on 500 ms trials (*M* = 0.93, SE 0.02) compared to 120 ms trials [*M* = 0.91, SE 0.02; *F*(1, 106.77) = 7.49, *p* < 0.01]. Load was not found to predict performance across the intervention period [*F*(1,104.14) = 0.32, *p* = 0.58].

#### Reaction time (RT; ms)

Significantly slower performance was observed on high load incongruent trials (*M* = 638.29, SE 26.64) compared to medium load incongruent trials [*M* = 614.52, SE 26.63; (*F*(2,104.30) = 4.50, *p* = 0.01] for placebo participants only (*p* = 0.01). This suggests that placebo participants performed worse on conditions of high demand. However, no such load differences were evident for WBB participants (high load incongruent *M* = 641.94, SE 25.27; medium load incongruent *M* = 641.27, SE 25.10), suggesting that this group’s RTs were unaffected, and potentially protected, from the manipulation of increased cognitive load.

As expected, from performance in previous cognitive studies [11–13], significantly quicker reaction times were evident for congruent trials (*M* = 607.50, SE 18.05) when compared to incongruent trials [*M* = 634.01, SE 18.06; *F*(1,112) = 19.88, *p* < 0.001]. There were no other significant predictors of MANT RT performance.

### AVLT

No treatment-related effects were observed on AVLT outcome measures.

### Mood

#### Positive (PA) and Negative (NA) Affect

Regardless of Drink, PA was lower at 4 weeks (*M* = 48.06, SE 1.94) compared to 2 weeks (*M* = 51.04, SE 1.94), an effect that narrowly missed achieving significance [*F*(1,15) = 4.27, *p* = 0.057]. No other significant results were observed for PA or NA measures.

#### Urinary polyphenol metabolites

Significant hippuric acid increases were observed after 2 week daily WBB supplementation. Changes in excretion also occurred for placebo participants, where concentrations of benzoic acid, 3-(4-hydroxyphenyl) propionic acid, syringic acid and isoferulic acid 3-*O*-β-d-glucuronide were significantly higher after 4 week daily placebo consumption.

A total of 27 phenolic metabolites were quantified in urine at weeks 0, 2 and 4 including pyrogallol, phenylacetic, valerolactone, benzoic acid, propionic acid, catechol, cinnamic acid and hippuric acid derivatives. Raw data for individual metabolite concentrations across weeks 0, 2 and 4 can be seen in Table 3.

**Table 3:** Mean (± SD) urinary polyphenol concentrations at week 0, week 2 and week 4 for placebo and WBB participants

	Urinary concentration (μg/24 h)					
	Placebo (*n* = 7)			WBB (*n* = 8)		
	Week 0 (*n* = 6)	Week 2 (*n* = 7)	Week 4 (*n* = 7)	Week 0 (*n* = 7)	Week 2 (*n* = 8)	Week 4 (*n* = 6)
Pyrogallol derivatives						
Pyrogallol-*O*-2-sulfate	3172 ± 1949	3043 ± 1745	2126 ± 964	5750 ± 6337	2807 ± 3595	3660 ± 4490
1-Methylpyrogallol-*O*-sulfate	941 ± 581	902 ± 452	673 ± 428	2424 ± 2457	1055 ± 1102	1599 ± 1635
Phenylacetic acid derivatives						
Homovanillic acid	522 ± 291	1406 ± 1660	626 ± 405	462 ± 310	624 ± 348	431 ± 224
3-hydroxyphenylacetic acid	57 ± 36	107 ± 50	126 ± 21	92 ± 68	139 ± 143	161 ± 102
4-hydroxyphenylacetic acid	651 ± 88	671 ± 294	778 ± 140	569 ± 250	671 ± 259	778 ± 291
Valerolactone derivatives						
(4R)-5-(3',4'-Dihydroxyphenyl)-gamma-valerolactone-4'-O-sulfate^b^	17 ± 38	67 ± 60	7 ± 6	3 ± 5	20 ± 23	25 ± 41
Benzoic acid derivatives						
Benzoic acid^a,b,*^	53 ± 14	52 ± 9	64 ± 13	48 ± 30	59 ± 18	52 ± 19
Protocatechuic acid^b^	616 ± 208	828 ± 284	662 ± 258	903 ± 592	577 ± 513	799 ± 790
2-hydroxybenzoic acid	0.02 ± 0.02	0.03 ± 0.04	0.03 ± 0.05	0.35 ± 0.40	0.23 ± 0.26	0.27 ± 0.31
4-hydroxybenzoic acid	7 ± 10	18 ± 32	13 ± 16	10 ± 12	12 ± 16	22 ± 26
Vanillic acid	646 ± 312	1152 ± 1420	846 ± 360	413 ± 227	393 ± 285	482 ± 282
4-METHYLGALLIC-3-*O*-sulfate	670 ± 356	778 ± 705	359 ± 225	994 ± 637	859 ± 1001	1813 ± 2746
2,4-dihydroxybenzoic acid	9 ± 7	8 ± 8	8 ± 5	8 ± 9	8 ± 4	7 ± 4
Syringic acid^b,*^	265 ± 284	393 ± 377	406 ± 237	87 ± 47	301 ± 270	220 ± 249
Propionic acid derivatives						
3-(4-hydroxyphenyl) propionic acid^b^	310 ± 133	340 ± 146	435 ± 83	235 ± 265	285 ± 205	175 ± 134
Catechol derivatives						
4-methylcatechol-*O*-sulfate	265 ± 156	346 ± 172	203 ± 44	164 ± 73	222 ± 84	171 ± 86
Cinnamic acid derivatives						
t-ferulic acid	51 ± 22	38 ± 18	44 ± 15	24 ± 13	28 ± 14	37 ± 23
Ferulic acid 4-O-sulfate	100 ± 150	439 ± 751	324 ± 544	28 ± 33	83 ± 115	71 ± 62
Ferulic acid 4-*O*-β-d-glucuronide	2114 ± 1671	2155 ± 962	3123 ± 2358	713 ± 691	600 ± 382	513 ± 349
Dihydro ferulic acid 4-*O*-β-d-glucuronide	7194 ± 7401	6879 ± 6718	4095 ± 2589	1214 ± 1001	1911 ± 1122	1310 ± 721
Isoferulic acid	134 ± 118	130 ± 95	168 ± 78	52 ± 49	49 ± 81	29 ± 22
Isoferulic acid 3-*O*-β-d-glucuronide^b^	673 ± 800	459 ± 284	689 ± 546	130 ± 161	126 ± 96	68 ± 59
Dihydro isoferulic acid 3-*O*-sulfate	2445 ± 2407	2281 ± 1462	2925 ± 2305	414 ± 338	513 ± 372	349 ± 411
Dihydro isoferulic acid 3-*O*-β-d-glucuronide	3692 ± 4801	7797 ± 4515	6578 ± 4864	917 ± 285	3176 ± 3079	2175 ± 1612
Dihydro caffeic acid 3-*O*-sulfate	13,987 ± 11,617	18,134 ± 12,800	10,451 ± 5701	6525 ± 7049	13,265 ± 13,497	19,219 ± 25,072
Chlorogenic acid	10,685 ± 23,737	214 ± 368	217 ± 300	1 ± 1	31 ± 45	58 ± 114
Hippuric acid derivatives						
Hippuric acid^a,*^	1226 ± 340	1171 ± 443	1158 ± 389	987 ± 394	1347 ± 262	1171 ± 262

*Significance at the *p* < 0.05 level for Time × Drink interactions. Details of within and between group significance is provided in Figs. 5, 6a–d and in Online Resource 1

^a^Metabolites that had higher concentrations after repeated WBB consumption

^b^Metabolites that had higher concentrations after repeated placebo consumption

There were no significant differences at baseline between treatment groups for total polyphenols excreted [*t*(11) = 1.39, *p* = 0.19]. Baseline variability appeared to be higher in the placebo group (SD 48,632) compared to the WBB group (SD 17,757); however, data were considered to be homogenous in a Levene’s test for equality of variances (*F* = 3.21, p = 0.1).

**Fig. 4:**
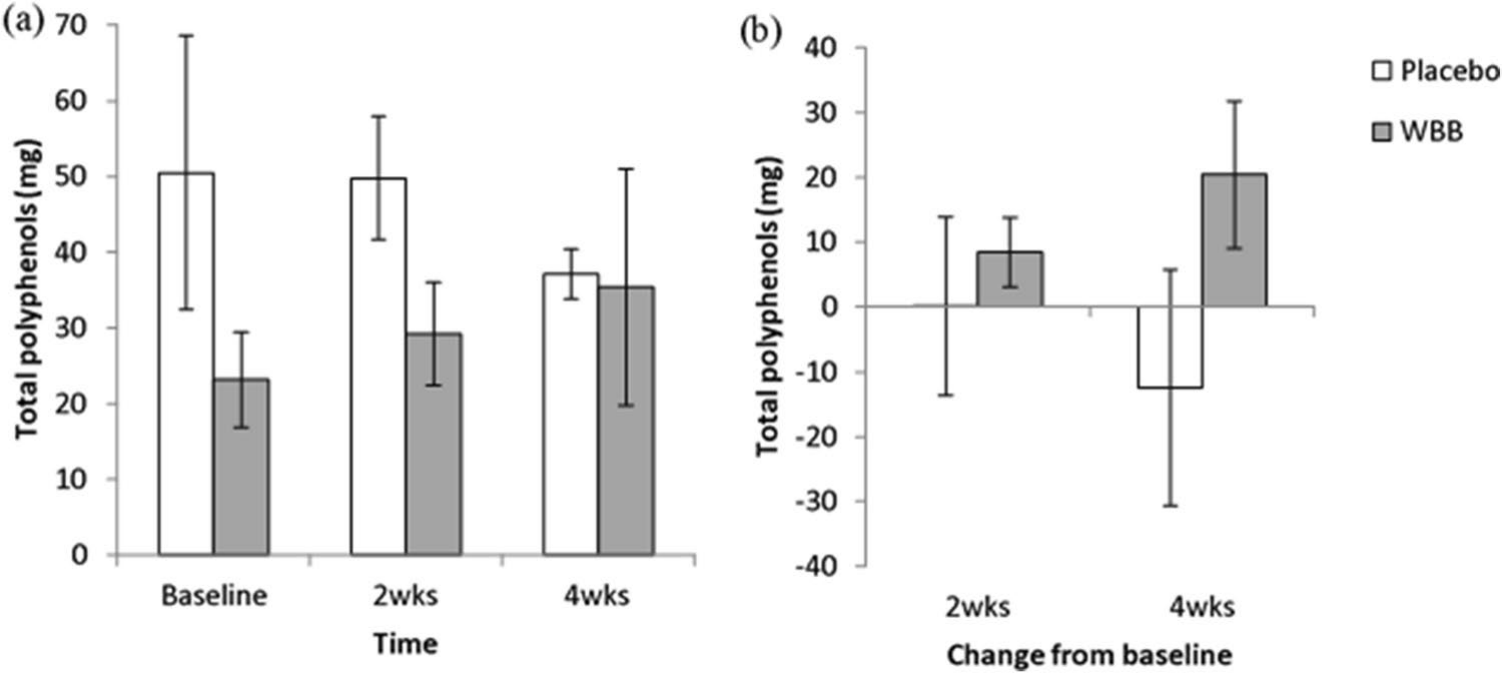
**a** Mean (± SEM) raw total polyphenol excretion at baseline, 2 weeks and 4 weeks for placebo and WBB participants, **b** mean (± SEM) total polyphenol change from baseline at 2 weeks and 4 weeks for placebo and WBB participants

As shown in Fig. 4a, analysis revealed no significant changes in excretion of polyphenols over the 4 weeks for either WBB [*F*(2,7.57) = 3.11, *p* = 0.10 or placebo *F*(2,7.23) = 1.64, *p* = 0.26], although there is a notable trend for an increase in polyphenol excretion following WBB treatment and a decrease in polyphenol excretion for those consuming the placebo treatment (Fig. 4b).

**Fig. 5:**
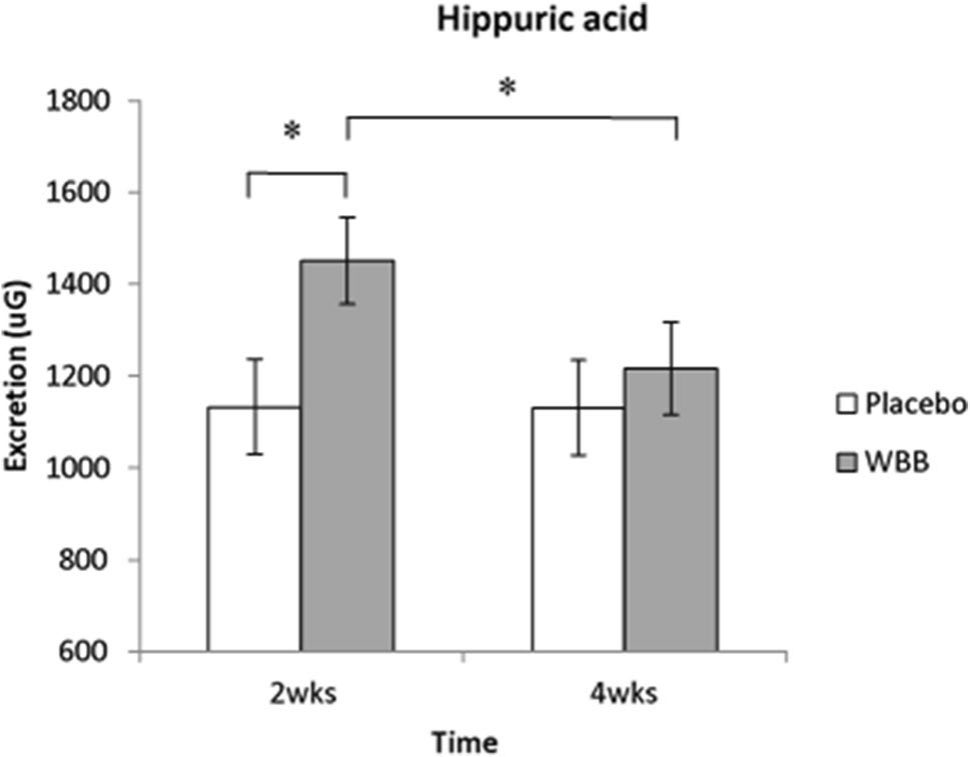
Significantly more hippuric acid was observed in WBB participants’ 24 h urinary excretion compared at 2 weeks compared to 4 weeks (*p* < 0.01) and compared to placebo participants (*p* = 0.04); *denotes significance at *p* < 0.05

Significantly higher concentrations of hippuric acid was observed at 2 weeks compared to 4 weeks in participants under a chronic WBB regimen [*F*(1,11.45) = 7.99, *p* = 0.016; Fig. 5].

**Fig. 6:**
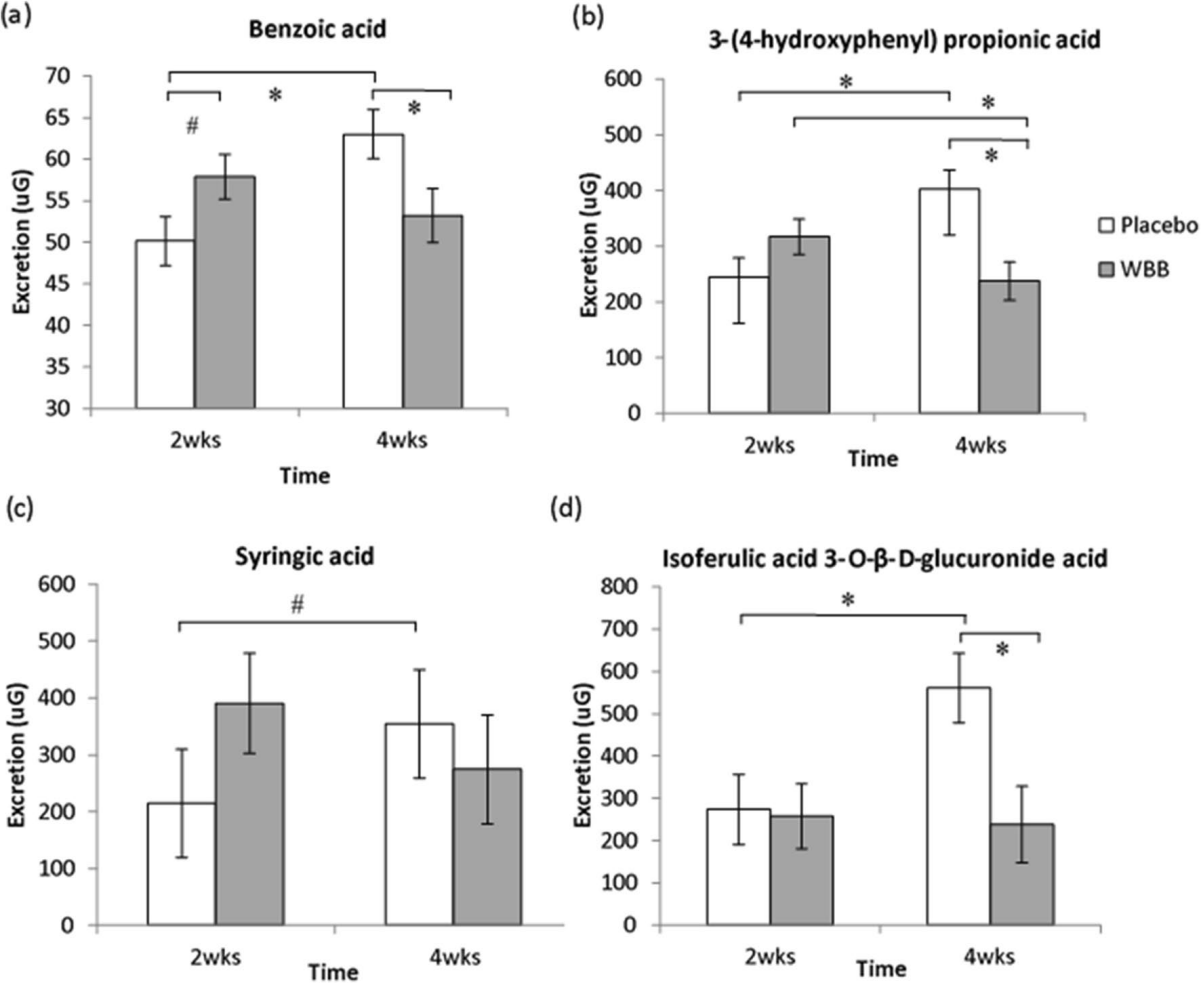
**a** Higher benzoic acid was seen for WBB participants at 2 weeks compared to placebo participants (*p* = 0.066). However, at 4 weeks, significantly higher excretion was observed in placebo participants in comparison to WBB participants (*p* = 0.038), and when compared to 2 week excretion levels (*p* < 0.01); **b** excretion of 3-(4-hydroxyphenyl) propionic acid was significantly higher at 4 weeks for placebo participants compared to 2 week excretion levels (< 0.01) and to WBB (*p* < 0.01). Excretion of this compound was found to significantly reduce from 2 to 4 weeks in participants under a chronic WBB regimen (*p* = 0.01); **c** higher excretion of syringic acid was observed at 4 weeks compared to 2 weeks for placebo participants (*p* = 0.06); **d** participants under placebo treatment excreted a significantly higher concentration of isoferulic acid 3-*O*-β-d-glucuronide at 4 weeks compared to 2 weeks (*p* = 0.018) and compared to WBB (*p* = 0.017)

Some metabolites showed a trend for an increase in the WBB group; higher concentrations of dihydro caffeic acid 3-*O*-sulfate were observed at 4 weeks compared to participants in the placebo group. Although a Drink × Time interaction was near-significant [*F*(1,11.79) = 4.45, *p* = 0.057], no further significant or trending post-hoc results were observed (Online Resource 1). Higher concentrations of benzoic acid [*F*(1,12.81) = 10.89, *p* < 0.01; Fig. 6a], 3-(4-hydroxyphenyl) propionic acid [*F*(1,11.69) = 43.78, *p* < 0.01; Fig. 6b], syringic acid [*F*(1,11.42) = 6.85, *p* = 0.02; Fig. 6c] and isoferulic acid 3-*O*-β-d-glucuronide [*F*(1,24) = 3.63, *p* = 0.069; Fig. 6d] were observed in placebo participants at week 4 compared to week 2.

## Discussion

The current pilot study administered a daily dose of flavonoid-rich wild blueberry to typically developing 7–10-year-old children for 4 weeks following a randomised, placebo-controlled, between-groups design, to investigate the effects of chronic consumption on mood, cognition and urinary metabolites.

Improvements in accuracy were observed on the MANT following WBB intervention, specifically on incongruent, fast and medium load trials. This suggests WBB was of most benefit to children under conditions of increased cognitive demand and on trials requiring higher speed of processing and alertness, as also observed on the MANT by Whyte et al. [13] and Barfoot et al. [15], respectively, in children aged 7–10. The current results do not match with Whyte et al.’s [14] recent findings in children in this age range, which revealed that WBB participants performed faster than placebo participants on congruent trials of an Attention Network Task (ANT) but not on incongruent trials. A reason this might be is that the ANT may not have been cognitively demanding enough to detect incongruent WBB effects in Whyte et al.’s [14] study. As the authors note, the ANT did not include the additional load and noise variables that are present in the MANT, potentially reducing the complexity of the task and sensitivity to blueberry effects.

Interestingly, when directly comparing congruent and incongruent MANT trials, performance was consistent for WBB, whereas performance significantly dropped off on incongruent trials following placebo. This suggests the WBB group did not succumb to manipulation of cognitive load. Children were able to maintain performance across easy and difficult trial types, which provides further support that WBB was of benefit to children under conditions of increased cognitive demand.

Such effects were also evident through manipulation of congruency and visual load. Placebo performed significantly slower on high load incongruent trials when compared to medium load incongruent trials. This was an expected effect of the task due to the increased cognitive resource required for high load trials. However, no such difference was observed for WBB, again suggesting maintenance of performance on the most cognitively demanding conditions of the task. These findings support Whyte et al.’s [13] and Barfoot et al.’s [15] research in children, which suggest supplementation with WBB can overcome the increased demand participants experience in conditions of high cognitive load, and specifically highlights the potential for WBB benefits across conditions of increasing complexity on the MANT, such as high load and incongruence. Furthermore, it is encouraging that similar WBB benefits have emerged in a smaller sample size than prior research at a lower power of 0.4 [*F*(2,26) = 3.37]. In addition, reassuring is that typical performance patterns on the MANT, regardless of treatment, were evident. Performance was significantly more accurate and faster on congruent trials compared to incongruent trials suggesting high internal validity for the task, irrespective of the small sample size. These observations indicate that the MANT may be sensitive at detecting WBB-related change on trials of higher difficulty using a lower number of participants than previously predicted. It is interesting, however, that typical load effects (worse performance on high load trials) did not occur regardless of drink. This questions whether the disparity between congruent and incongruent trials is larger than that between load trials and whether incongruent trials are more difficult than trials of high, medium and low load. This may be why load effects did not persist in this small sample. WBB participants performance was maintained on the more difficult high load, incongruent trials suggesting that in combination, the required complexity to detect a WBB effect was potentially achieved. Future studies might like to assess the difficulty of MANT trial type combinations to see if WBB is of most benefit on the most complex trials.

Accuracy was revealed to be significantly higher in the WBB group compared to the placebo group on 120 ms trials. In addition, accuracy did not differ between fast (120 ms) and slow (500 ms) trials of the MANT for WBB participants, as opposed to placebo participants whose accuracy significantly dropped on fast trials. These findings indicate WBB participants were able to perform more accurately on faster paced trials across a 4 week WBB supplementation. Interestingly, a significant Drink × Target Time interaction also occurred in children in Barfoot et al. [15], where significantly quicker RT occurred on the faster 120 ms trials after acute (2 h) WBB supplementation, at no cost to accuracy. This indicated improved speed of processing and mental alertness on faster trials under an acute design. It is interesting that WBB RT benefits occurred after a one-off dose, whereas WBB accuracy was higher under a 4 week regimen, suggesting different facets of executive function (including time to process, ability to integrate information to make a correct response and mental alertness) may respond differently to acute and chronic WBB interventions.

Overall, a lack of treatment-related findings were observed for mood and memory outcomes (PANAS-C and AVLT, respectively). Previous effects observed on these measures in children, using parallel group designs were in larger samples (AVLT, n = 54 [15]; PA, n = 54 [17]); therefore, the small sample size could explain absence of treatment effects here for these tasks. In addition, the WBB benefits observed in the aforementioned research occurred in acute trials, and futher work is needed to explore memory and mood in children following chronic WBB supplementation. In the present study, positive affect was found to significantly decline at 4 weeks compared to 2 weeks, regardless of treatment. This could be explained by increased feelings of tedium and repetitiveness as the novelty of the experiment diminished over time. It is important to note that if this was the case, these feelings did not influence negative affect as these scores did not change over the intervention period.

Increased excretion of total polyphenols was observed for WBB participants over the course of the intervention, but this did not reach statistical significance, whilst placebo participants showed a non-significant decline. This implies that repeated daily consumption of a WBB drink across 4 weeks may increase the total concentration of polyphenolic metabolites excreted in 7–10-year-old children, but a larger sample is required to confirm the subtle indications from this pilot. Specifically, the degree to which changes in total metabolites are driven by individual metabolites in children following WBB requires further investigation, as the present study provided limiting findings regarding this from 27 compounds.

Here, higher concentrations of hippuric acid were observed at 2 weeks following WBB relative to 4 weeks. As per a-priori statistical methods, comparisons were not made directly with baseline, rather baseline was included in the model as a covariate (LMM fixed factor). Previous research has shown changes in hippuric acid following WBB interventions. Rodriguez-Mateos et al. [33] observed a rise in plasma hippuric acids across 4 weeks of freeze-dried WBB intervention in healthy adults (22 g/day; containing 300 mg/day anthocyanins). Interestingly, vascular improvements (FMD) occurred after 1 week of daily supplementation, rising further after 2 weeks, but plateauing between 2 and 4 weeks and it was postulated that 2 weeks may be an optimal timeframe to sustain beneficial endothelial change. High concentrations of hippuric acid following 2 weeks of daily WBB in the current study partly supports this theory. It would be of interest to explore whether metabolites may mediate vascular and cognitive changes specifically at the 2 week timepoint in children. Similarly, Feliciano et al. [21] reported that hippuric acid was found to be the largest contributor (~ 86%) to the pool of circulating metabolites in plasma following 30 day WBB supplementation in adults. Such increases in hippuric acid may be due to interactions between blueberry conjugates and gut microbiota. Indeed, hippuric acid is a metabolite that is derived from gut microbial metabolism. The efficiency of the gut to metabolise polyphenols may have, therefore, been positively affected and this warrants further exploration. Interestingly in Feliciano et al.’s [21] study, adult concentrations of hippuric acid were much higher at day 0 (M = 55,827 ± SEM = 7000) and day 30 (M = 62,840 ± SEM = 8552) following the WBB regimen compared to child concentrations in the current study (day 0, M = 987 ± SEM = 394; day 30, M = 1171 ± SEM = 262). Concentrations were similarly low in children following the placebo regimen in the current study (day 0, M = 1226 ± 340; day 30, M = 1158 ± 389). This suggests that children may have lower absorption rates than adults and requires further investigation. It is important to acknowledge that the current study and Feliciano et al.’s study did not employ the same methodology, so comparisons between participant concentrations across studies must be interpreted with caution. Similar increases in hippuric acid in adult cohorts have been observed in plasma following a 12 week daily bilberry intervention [42], and in urine after an 8 week high-polyphenol diet intervention [43]. This highlights hippuric acid as a potential metabolite of interest when considering the chronic improvements that have been observed in health [44] and cognitive domains [6–10].

At present, a best practice for converging metabolite and cognitive data is yet to be established. It is vital that nutritional interventions investigating physiological or cognitive measures include a placebo control; however, the method by which treatment and placebo data are analysed currently differs. Metabolomic comparisons are often made within-participants, identifying compounds that have changed across time for individuals in each treatment group. As metabolite variability is high across individuals and dependent upon many variables (e.g., diet, genetics), between-group comparisons would not account for such variability and would provide an unreliable estimate of treatment effect. On the other hand, cognitive comparisons need to occur within and between treatment groups to examine changes across time and in comparison to a similarly performing control group. It is well known that large inter-individual variation exists between individuals across investigations into polyphenol metabolism (for review see [45]). Genetic variability in the expression of metabolic enzymes and transporters is thought to play a part in post-absorptive metabolism, and these metabolic functions are likely to work differently depending on the individual, food source and dose [46]. High inter-individual variability is, therefore, expected in investigations of this kind, highlighting the importance of within-participant comparisons. Such individual variation may account for the lack of significant increases in some metabolites observed between 2 and 4 weeks. Future research should consider employing a chronic, within-groups, crossover design (with an appropriate washout period) so that cognitive and metabolite data can be assessed in the same participant sample using placebo and WBB arms.

It is feasible that flavonoids derived from other dietary sources in the 48 h prior to the 4 week urine collection, may have converged with the flavonoids present in WBB, producing increased excretion of metabolite compounds. From 3 day food diary data, participants can be said to have average fruit and vegetable consumption when compared to the latest Health Survey for England [47] which shows children aged 5–15 consume ~ 3.1 portions of fruit and vegetables a day. It could, therefore, be possible that children consumed an average amount of fruits and vegetables in the days prior to urine collections, leading to interactions between blueberry and other flavonoids.

Similarly, other dietary constituents may have interfered with metabolite excretion in the placebo group, where benzoic acid, 3-(4-hydroxyphenyl) propionic acid, syringic acid and isoferulic acid 3-O-β-D-glucuronide excretion increased at 4 weeks. Indeed, isoferulic acid is found in many low flavonoid dietary sources, such as peanuts, wheat [48] and pineapples [49], and benzoic acid is often used as a preservative in cordial squash. Although participants were asked to follow a low flavonoid diet for 48 h prior to urine collections, they were still permitted to consume foods deemed ‘low flavonoid’ that may have produced high levels of polyphenolic metabolite compounds. It may, therefore, be of use in the future to derive a standardised low polyphenol diet based on high polyphenolic compound levels rather than high flavonoid food status alone. It is also important to consider the necessity of adding squash to both WBB and placebo treatment drinks. Rock’s squash contains small amounts of organic acids [50] which may interfere with metabolite levels directly or interact with other dietary constituents to produce confounded levels. In the current and previous studies, Rock’s squash has been used for palatability purposes. Exploration of other low polyphenol additions to increase palatability of placebo and WBB drinks is, therefore, warranted.

Interestingly, placebo participants failed to respond to non-target stimuli at a significantly higher rate than WBB participants on the demographic measure of sustained attention. This suggests that those assigned to the placebo group were, by chance, of a lower attentional ability. This was supported by significantly lower accuracy on the MANT for this group at baseline, a task requiring attention. However, MANT accuracy was consistently high in both placebo (range = 80.75–88.52%) and WBB (range = 92.38–96.82%) groups across the course of the intervention, suggesting this characteristic may not have been a detriment to placebo participants beyond baseline. Indeed, no significant differences were observed between treatment groups at the 2 or 4 week timepoint when including baseline as a fixed factor in the LMM.

An additional limitation is that the placebo drink was rated as significantly more likeable compared to the WBB drink. This could be due to the texture of the WBB drink, which had a somewhat grainier consistency. Future studies should consider including other palatability measures, such as texture and smell to determine aspects of the drinks that participants do not like, to further inform adequate placebo-control matching. The important aspect of the current study is that no participant rated their assigned treatment drink below a 4 (out of 10), indicating their treatment was drinkable. Importantly, this finding suggests that WBB-related improvements are not due to high drink likeability.

Future research should build on these initial findings by converging cognitive effects with metabolite excretion and a measure of cerebral blood flow (CBF), such as arterial spin labelling (ASL), regional functional magnetic resonance imaging (fMRI), or magnetoencephalography (MEG), to better inform the field of a chronic mechanism of action (MOA). Investigation into how polyphenolic metabolites enter the BBB and impact the brain is still in its’ infancy; however, a promising review by Carregosa et al. [51] has identified mechanisms by which low-molecular weight polyphenol metabolites, such as hippuric acid, may attenuate neuroinflammation via action within the central nervous system’s microglia cells. These immune cells are responsible for the surveillance and action on neurotoxic and neuroprotective processes, rendering them key to brain repair, renewal and disease progression. Investigation of the aforementioned neuropsychological and molecular techniques alongside measures of cognitive performance would permit measurement of the specific flavonoid metabolites that are circulating in the body when cognitive effects are observed, and would elucidate whether regional CBF effects are also present across the same time frame. This would allow researchers to postulate the specific flavonoid metabolites that may be able to cross the BBB and exert vasodilatory effects on the brain in the presence or absence of cognitive improvements.

The current pilot study aimed to measure the cognitive ability and urinary metabolite concentrations of healthy 7–10 years following a 4 week daily WBB regimen. It was predicted that cognition may improve following WBB supplementation. Significant improvements in cognition were observed in children consuming WBB, specifically on trials requiring high cognitive demand, as has been seen in previous child acute WBB research [13]. WBB-related memory or mood effects were not evident, despite the previous benefits that transpired in acute child trials [15, 17]. The study also aimed to explore urinary excretion levels in a child cohort under a chronic WBB design for the first time. Hippuric acid levels were found to be significantly higher at 2 weeks, aligning with previous chronic adult berry [21, 42] and polyphenol-rich diet [43] research. Children may excrete polyphenol derivatives in a similar pattern to adults and hippuric acid may be a potential metabolite of interest when considering health and cognitive benefits across adult and child populations. However, further work is required to elucidate the specific metabolomic processes occurring in a child population across chronic timeframes under WBB and placebo interventions. Specifically, research should aim to investigate metabolite levels, cognitive performance and endothelium-related change in conjunction to try and elucidate the MOA behind WBB benefits.

## Supplementary Information

Below is the link to the electronic supplementary material.

Supplementary file1 (PDF 125 kb)

## Data Availability

All data, materials and software application support published claims and comply with field standards.
