# Supplementary material for: Effects of daily consumption of wild blueberry on cognition and urinary metabolites in school-aged children: a pilot study
Source: Eur J Nutr. 2021 May 23;60(8):4263–78. doi: 10.1007/s00394-021-02588-y (PMC8572198; doi:10.1007/s00394-021-02588-y)
Supplement: Supplementary file 1 — Supplementary file1 (PDF 125 kb) [file 394_2021_2588_MOESM1_ESM.pdf]

Online Resource 1

Article title: Effects of daily consumption of wild blueberry on cognition and urinary metabolites in school-aged children: a pilot study

Journal name: European Journal of Nutrition

Authors: Barfoot, K.L., Istas, G., Feliciano, R.P., Lamport, D.J., Riddell, P.M., Rodriguez-Mateos, A. & Williams, C.M.

Correspondence: Professor Claire Williams; claire.williams@reading.ac.uk; School of Psychology and Clinical Language Sciences, University of Reading, Earley Gate, Whiteknights Road, Reading, UK

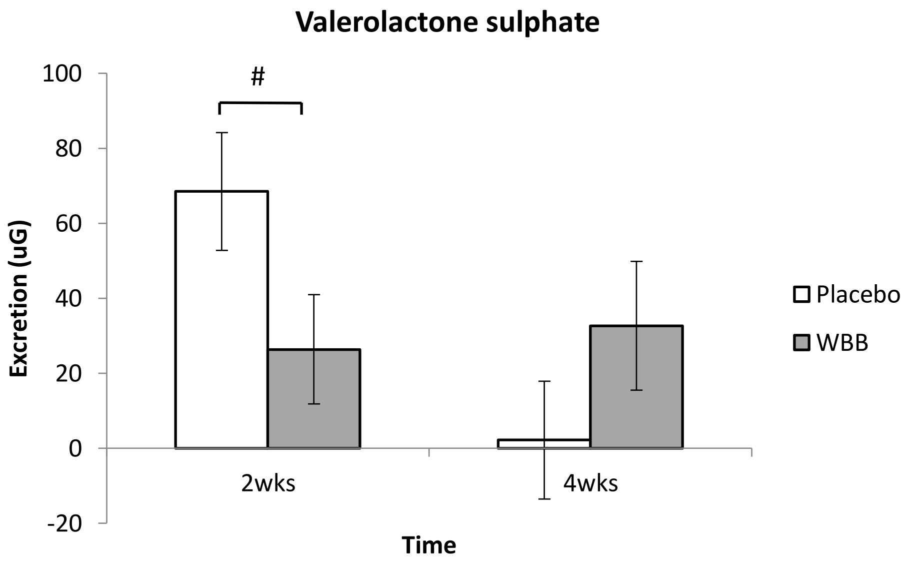

Fig. 1 Placebo participants excreted higher concentrations of valerolactone sulphate than WBB participants at the 2 week time point (p=0.06; F(1,24)=5.36, p=0.029)

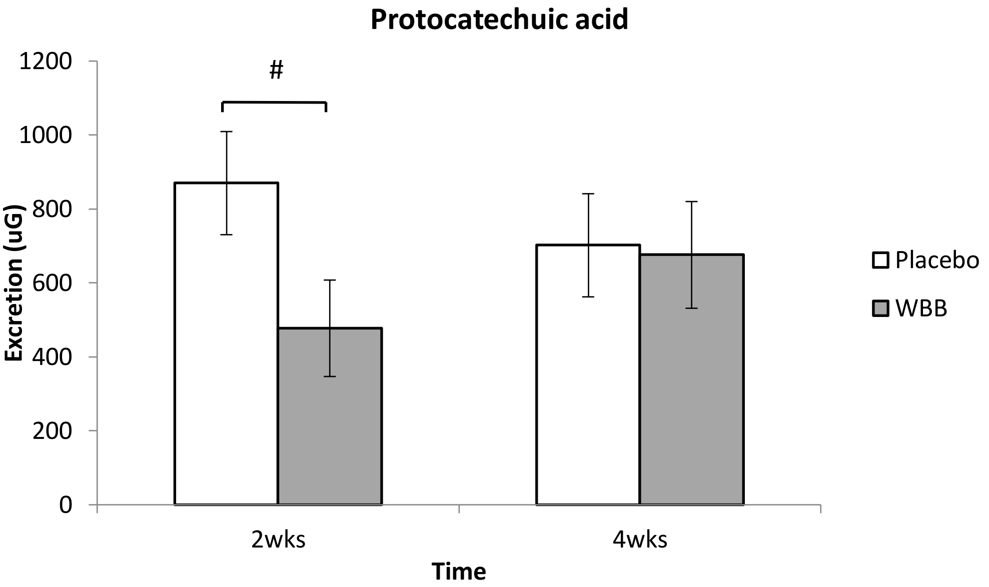

Fig. 2 Placebo participants excreted higher concentrations of protocatechuic acid than WBB participants at the 2 week time point (p=0.06; F(1,8.14)=4.30, p=0.07)

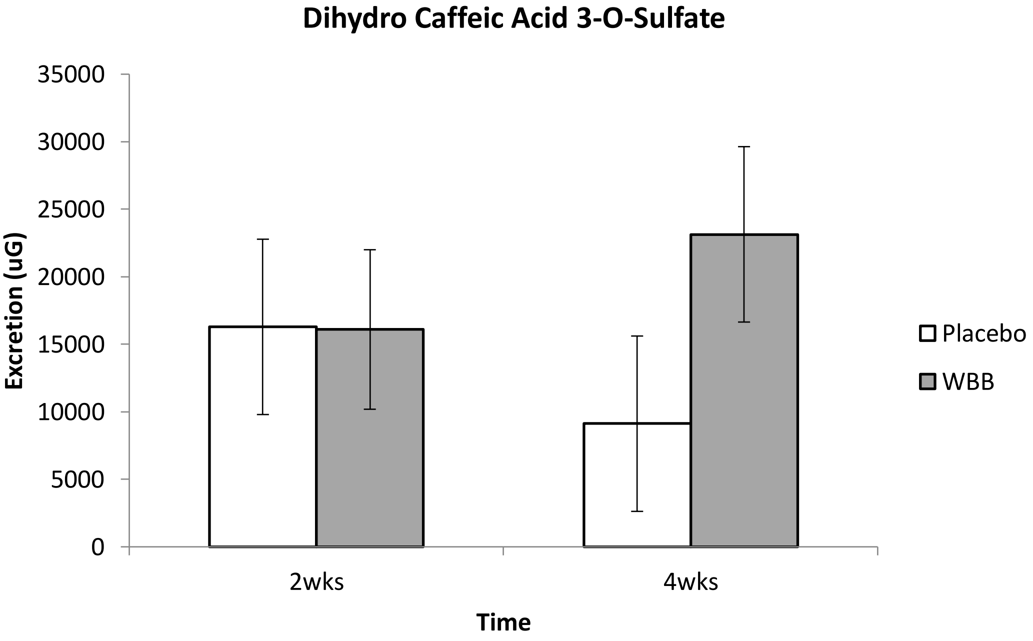

Fig. 3 Participants consuming WBB appear to have higher concentrations of dihydro caffeic acid 3-O-sulfate at 4 weeks compared to participants consuming placebo. Although a Drink x Time interaction was near-significant (F(1,11.79)=4.45, p=0.057), no further significant or trending post-hoc results were observed
